# GABA inhibits proliferation and self-renewal of mouse retinal progenitor cell

**DOI:** 10.1038/s41420-019-0160-z

**Published:** 2019-03-22

**Authors:** Shaojun Wang, Lu Du, Guanghua Peng, Wei Li

**Affiliations:** 1 Department of Ophthalmology, General Hospital of Chinese People’s Liberation Army, Beijing, 100853 China; 2 Department of Ophthalmology, Affiliated Hospital of Academy of Military Medical Sciences, Beijing, 100071 China; 3 Department of Obstetrics and Gynecology, Tongji Hospital, Tongji Medical College, Huazhong University of Science and Technology, Wuhan, 430030 Hubei China

## Abstract

Gamma-amino butyric acid (GABA) is the main inhibitory neurotransmitter in the central nervous system, including the retina, and play an important role in both regulating neurogenesis and neural stem cell proliferation. GABAa receptor has been identified in the retina, however, the function of GABAa receptor on retinal progenitor cell (RPC) is unclear. RPCs were cultured to analyze changes in cell proliferation and cell cycle distribution after GABAa receptor activation. The activation of GABAa receptor significantly inhibits RPCs proliferation, cell cycle progress, and self-renewal. Moreover, the activation of GABAa receptor leads to the up-expression of p21 and p27 and down-expression of Nestin, Pax6, Sox2, and Chx10. These results suggest that GABA acts as a negative regulator of RPCs proliferation and self-renewal.

## Introduction

Retinal degeneration diseases, such as retinitis pigmentosa and age-related macular degeneration, which are characterized by photoreceptor degeneration and death, often result in complete vision loss^1^. Both bench and clinical trials showed that transplantation of stem cell is a promising therapy for treating the retinal dysfunction by replacing the damaged cells^2–4^. It has been demonstrated that extracellular signals, such as growth factors and neurotransmitters, could affect the proliferation, self-renewal, and differentiation of stem cells^5^. In vivo study provided evidence that microenvironment inhibits proliferation of grafted stem cells^6^. A lot of neurotransmitters, such as glutamine and γ-amino butyric acid (GABA), exist in the microenvironment of the retina; it is important to study the mechanisms for controlling the proliferation and self-renewal of the retinal progenitor cells (RPCs) by neurotransmitter^7,8^.

GABA is one of the main inhibitory neurotransmitters in the central nervous system, including the retina^9,10^. Besides neural information processing, GABA is involved in regulating neurogenesis^11,12^, such as proliferation, differentiation, and migration of neural stem cells (NSCs)^13–16^. Song et al. have pointed out that GABA regulates hippocampal neurogenesis and neuronal development^17,18^. Subsequently, Song et al. found GABA could directly affect NSCs, and decreased the number and proportion of proliferating NSCs in the dentate gyrus^19^. Interestingly, they also showed local interneurons could regulate neurogenesis in the distal region through GABA signal pathway^12^. Moreover, the role of GABA in stem cell regulation is not restricted to the hippocampus, it has been identified as a negative regulator of stem cell proliferation in a number of other contexts, including the embryonic stem cell and spermatogonial stem cells^20–23^. All these results indicated that GABA is an important niche factor to maintain stem cell pool homeostasis in vivo^11,24^.

Although functional GABAa receptor has been identified in RPCs^25^, it is not known whether GABA could regulate proliferation and self-renewal of RPCs. Identifying the mechanisms that underlie RPC proliferation and self-renewal will enhance our understanding of retinogenesis during embryonic development, and, more broadly, reveal stem cell biological principles extending to tissue regeneration. So, the aim of our present work is to address this issue and explore the molecular mechanism of GABA on RPCs proliferation and self-renewal.

## Results

### Characterization of primary cultured RPCs

**Fig. 1:**
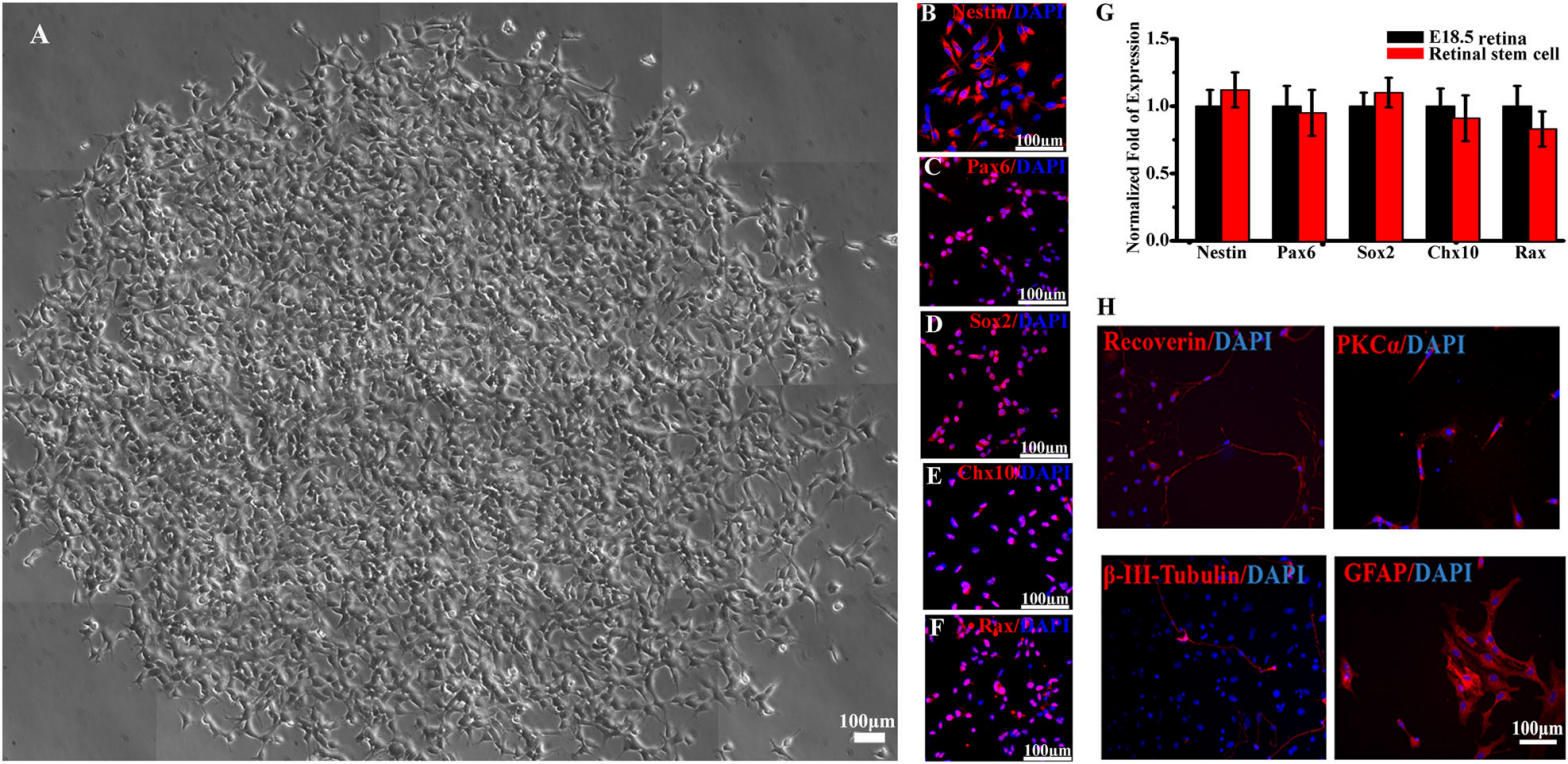
RPCs were isolated from adult retina. **a** Phase-contrast imaging of a representative RPC clone from single cell. **b**–**f** Cells express high levels of RPC markers, Nestin (**b**); Pax6 (**c**); Sox2 (**d**); Chx10 (**e**); Rax (**f**). **g** Cells express mRNA transcripts of RPC markers: Nestin, Pax6, Sox2, Chx10, and Rax. mRNA expression levels were compared between RPCs and E18.5 retina tissue by real-time quantitative RT-PCR analysis and GAPDH was used as an internal control. **h** Representative images of immunostaining for recoverin, PKCa, β-III-Tubulin, and GFAP. (*p < 0.05 vs control, *n* = 3)

Adult mice retina was digested into single cell and plated on the dish coated with gelatin. Only a few cells attached to the dish and grew in heterogeneous morphology. After 3 passages, we seeded 500 of the cell on the ⌀150 mm dish. Most of these cells lost their proliferative ability after passage. Ten days later, only several spindle-shaped small cells could form homogeneous clones in the dish (Fig. 1a). We picked up 5 clones from each dish with a small filter paper with enzyme. Then the cells were amplified singly, cells from each clone could proliferate stably with homogeneous morphology. These cells can be cultured in vitro for at least 5 months (over passage 35), passaged every 3–5 days. We repeated three times and got 15 clones of the retinal stem-like cells. Immunostaining showed that the retinal stem-like cells expressed the RPCs marker, Nestin, Pax6, Sox2, Chx10, and Rax (Fig. 1a). Then, we compared the expression of these stem cell markers with embryonic 18.5 mouse retina. Real-time PCR analysis showed there is no obvious difference of the Nestin, Pax6, Sox2, Chx10, and Rax between the two samples. The RPCs could be differentiated to photoreceptor cells, ganglion cells, bipolar cells, and Muller glial cells (Fig. 1h).

### GABA inhibits proliferation of RPCs, not affects cell survival

**Fig. 2:**
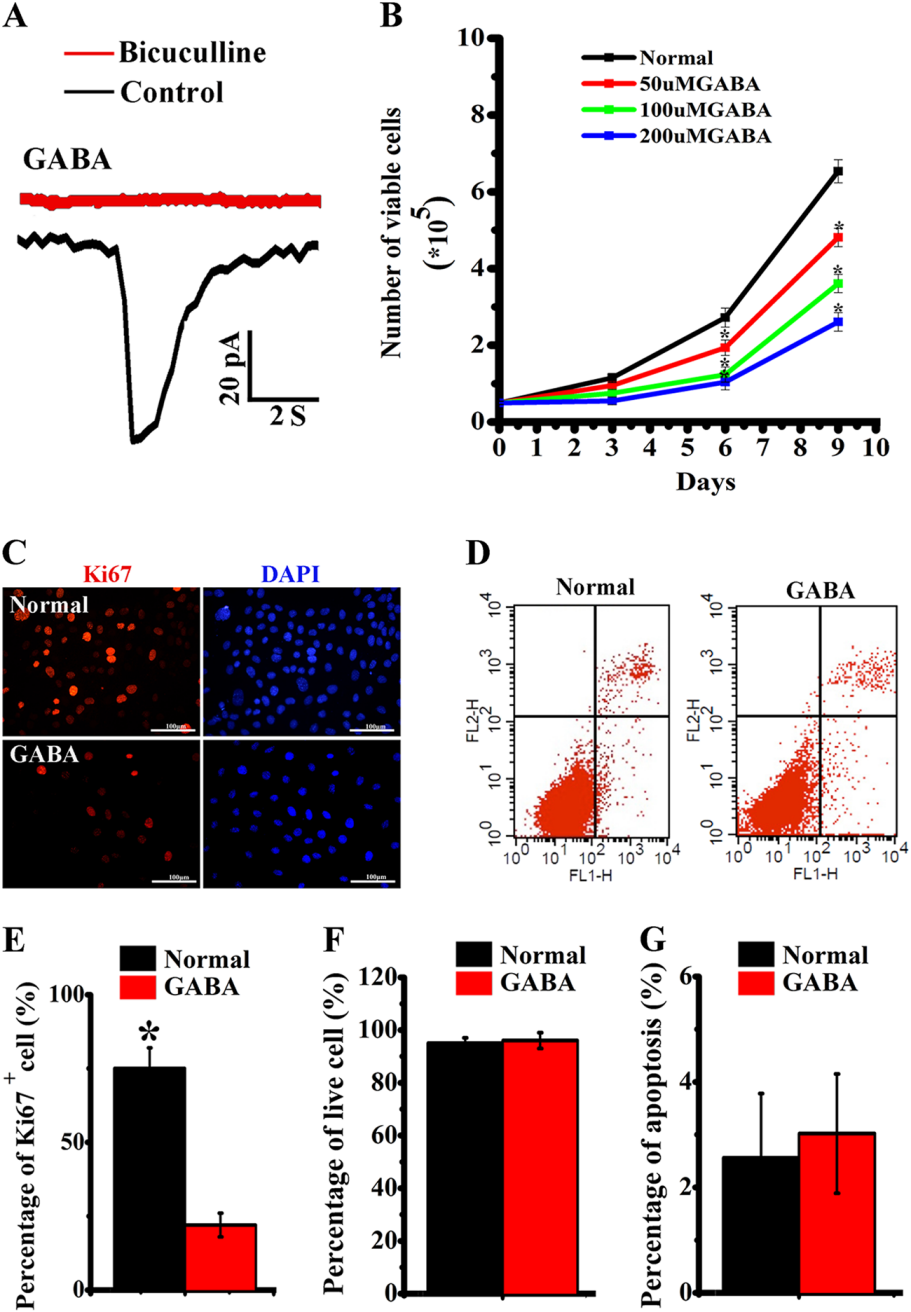
Effects of GABA stimulation on the proliferation and viability of RPCs. **a** Electrophysiological recording of GABA stimulating evoked inward currents in RPCs in voltage clamp mode, and abolished by GABAaR antagonist bicuculline. **b** Different concentration of GABA treatment suppressed the proliferation curve of RPCs. **c**–**e** Expression of Ki67 after a 48-h treatment of 100 µM GABA stimulation. Immunostaining images showed the downregulated expression of Ki67 in RPCs after treatment with GABA (**c**); flow cytometry graph of apoptosis after GABA treatment (**d**); histogram chart showing the expression of Ki67 after GABA stimulation (**e**). **f**, **g** Histogram showing that GABA stimulation did not affect the viability (**f**) and apoptosis ratio (**g**) of RPCs. (*p < 0.05 vs control, *n* = 3)

GABA or the GABAaR-specific agonist muscimol (data not shown) evoked an inward current in whole-cell voltage-clamp recordings of RPCs (*n* = 15) and the current was attenuated significantly by the GABAaR-specific antagonist bicuculline (100 µM) (Fig. 2a). Subsequently, we tested if the activation of GABA signaling pathway affects the proliferation of RPCs. Application of 50, 100, or 200 µM GABA obviously decreased the proliferation of RPCs compared with control (Fig. 2b). Subsequently, we applied 100 µM GABA to assay the expression of cell division marker Ki67. We observed that the percentage of Ki67-positive cells in the GABA treatment group was significantly lower than the control group (Fig. 2c–e). We then tested whether the decrease in cell number was due to differences in apoptosis or in proliferative rates. Percentage of viable cells was analyzed by the trypan blue exclusion assay, and GABA treatment did not affect RPCs viability (Fig. 2f). Additionally, the flow cytometry data of apoptosis showed almost no apoptotic cells in either GABA treatment or control group (Fig. 2g).

### GABA treatment causes G1 phase accumulation and S phase reduction

**Fig. 3:**
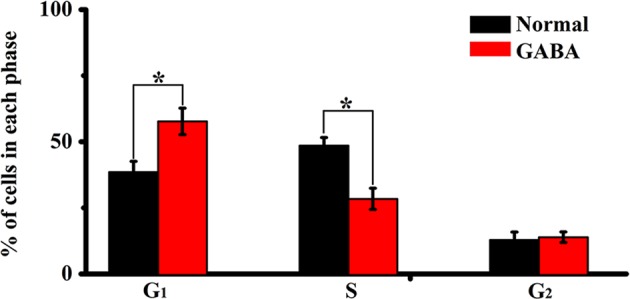
Effect of GABA stimulation on the cell cycle distribution of RPCs. GABA increased the percentage of cells in G_0_/G_1_ phase and decreased the percentage of cells in S phase. (*p < 0.05 vs control, *n* = 3)

The cell cycle distribution of RPCs after GABA treatment is summarized in Fig. 3. Application of 100 µM GABA resulted in a significant decrease in the proportion of cells in S phase and increase of cells in G_0_/G_1_ phase. The percentage of cells in G_0_/G_1_ phase in the control group was 38.57 ± 4%, GABA increased the percentage to 52.69 ± 5% (*p* < 0.05 vs control). In addition, the proportion of cells in S phase decreased from 48.56 ± 3% in control to 33.4 ± 4% in GABA treatment group (*p* < 0.05 vs control) (Fig. 3).

### GABA inhibits self-renewal of RPCs

**Fig. 4:**
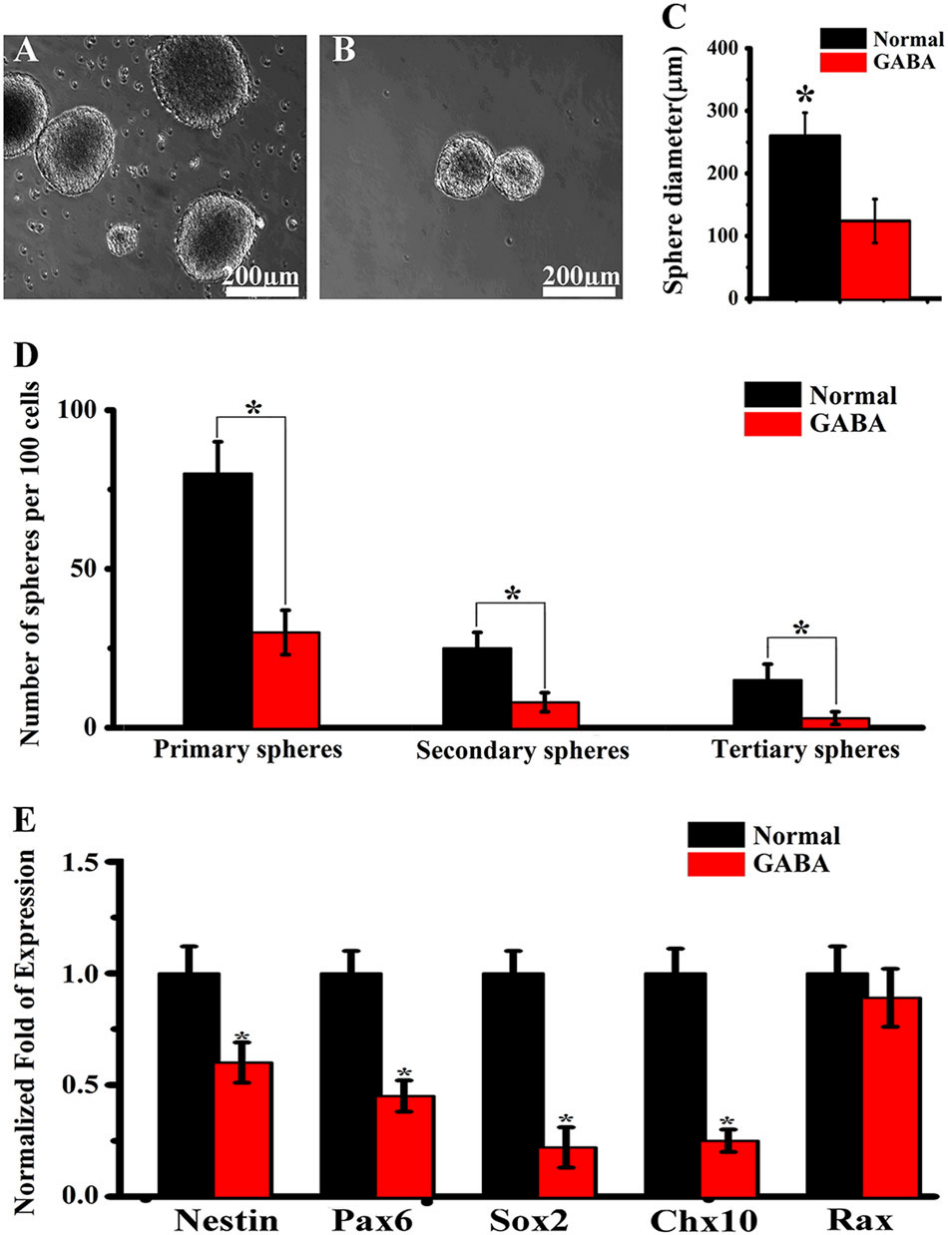
The self-renewal ability of retinal progenitor cells was inhibited by GABA treatment. **a**, **b** Sample images of control and GABA treatment group neurosphere. **c** The diameter of primary neurosphere generated from GABA group is significantly smaller than the control. **d** The self-renewal ability of retinal progenitor cells culture with or without GABA treatment assessed by secondary and tertiary neurosphere formation. **e** Real-time quantitative PCR analysis of the Nestin, Pax6, Sox2, Chx10, and Rax gene expression level of retinal progenitor cell after GABA treatment. (*p < 0.05 vs control, *n* = 3)

To investigate the role of GABA signaling in regulating self-renewal of RPCs, we performed neurosphere assay. The volume of neurosphere from GABA treatment group was significantly smaller than the control. The average diameter of the GABA treatment was 129 µm, and the control group neurosphere was 264 µm (Fig. 4a–c). Neurosphere size is a rough parameter for self-renewal potential of RPCs. The growth curve of the neurosphere from multiple generations is a more accurate parameter for the self-renewal capabilities of the RPCs. Subsequently, we performed sphere-forming assay by analyzing the percentage of the cells that formed neurosphere at each consecutive passage. The percentage of the sphere-forming cells was 2.7-fold decreased in the GABA treatment group compared with the control group at passages 1 and 2, respectively (Fig. 4d). The real-time PCR results showed after GABA treatment, the Nestin, Pax6, Chx10, and Sox2 gene expression level, was significantly down-regulated (Fig. 4e), which might be the cause of inhibited self-renewal. These findings demonstrate an obvious inhibition effect of GABA signaling pathway in modulating self-renewal of the RPCs.

### GABA inhibits proliferation and self-renewal of RPCs through inducing p21 and p27

**Fig. 5:**
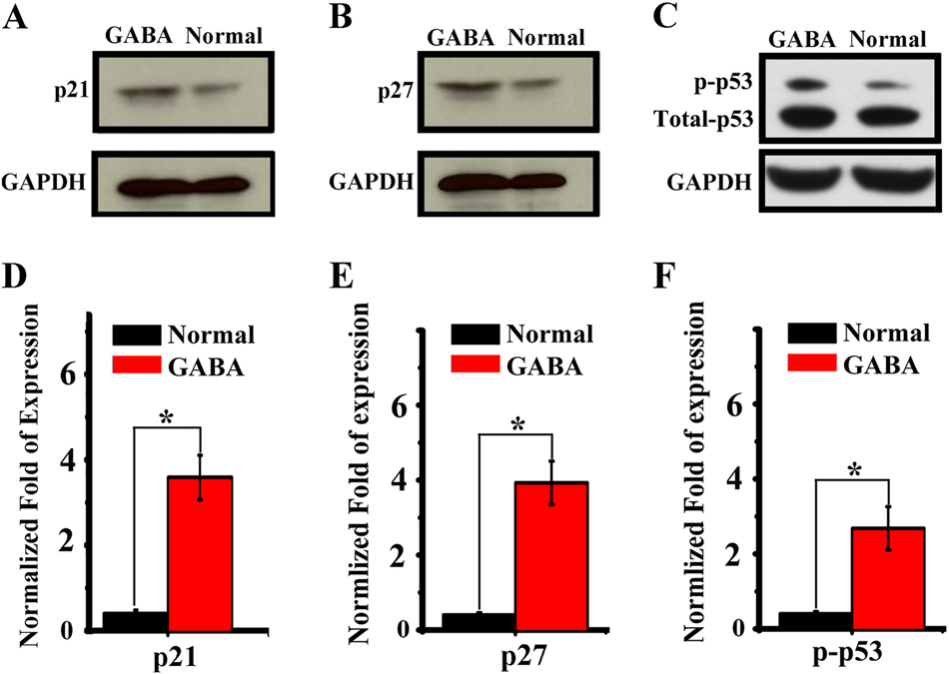
Effect of GABA stimulation on the cell cycle regulator of retinal progenitor cells. **a**–**c** Western-blot analysis of the p21, p27, p53 protein expression level of retinal progenitor cell in control and GABA treatment groups independently. The gels in this experiment were run under the same experimental conditions. **d**–**f** Histogram chart showing the expression of p21, p27, p53 and p53 phosphorylation after GABA stimulation. (*p < 0.05 vs control, *n* = 3)

The results above showed that GABA could substantially prevent the proliferation and self-renewal of RPCs. Next, we explored the change of molecular signal involving in cell cycle in RPCs. We detected the expression level of p27^Kip1^ and p21^CIP1^, which are two members of the Kip/CIP family known to regulate cell proliferation and terminal differentiation in a variety of cell types. The Western-blot results showed when treated with GABA, the p27^Kip1^ and p21^CIP1^ levels were significantly up-regulated. We also assayed the expression level of p53 after GABA treatment, and found the up-regulation of phosphorylated p53, while the total p53 has no obvious change (Fig. 5).

## Discussion

The present work illustrates that GABA signaling could affect proliferation and self-renewal of RPCs. Furthermore, the activation of GABAa receptor could modify the p21, p27 and Nestin, Pax6, Sox2, Chx10 level, and lead to the inhibition of proliferation and self-renewal of RPCs.

Stem cell-based cell therapy is promising for the retinal degeneration disease. Illustrating the mechanisms involved in RPCs proliferation and self-renewal will boost stem cell-based therapies for retinal degenerative diseases toward the clinic. Self-renewal or differentiation of RPCs is controlled by certain circumstances, or niches^26^. GABA signaling represents one niche mechanism that regulates adult neurogenesis^12,19^. GABA has been shown to decrease the proliferation of other stem cells and progenitors in vitro, including mouse embryonic stem cells and spermatogonial stem cells^20–23^. Moreover, one study has found GABA treatment could induce beta-like cell of islet neogenesis from duct precursor cells^27^.

The previous study has identified GABAa receptor in RPCs isolated from ciliary body margin, however, the cell isolation and culture is different, and whether GABA could affect the self-renewal or differentiation is not fully illustrated. In our work, we have isolated the RPCs from the adult mouse neural retina tissues (not from ciliary body margin) and keep the self-renewal and multipotency in long time culture. Furthermore, we found the activation of GABAa receptor could affect the proliferation and self-renewal of RPCs in vitro. In our further work, we need to investigate the regulation of GABA on the RPCs in vivo both in normal and pathological conditions. Furthermore, we will explore whether the RPCs proliferation and self-renewal could be inhibited by GABA after transplant in the retina in vivo, and explore if the repair efficiency could be enhanced by block GABAa receptor of RPCs before transplant.

One highly conserved signal that controls cell development in both neural and peripheral stem cell niches is the neurotransmitter GABA acting through GABAa receptors^28,29^. A few groups have undertaken the task of exploring the role of GABA in mediating both developmental and adult neurogenesis^11,12,18,30^. The inhibitory neurotransmitter GABA had previously been suggested to regulate several aspects of hippocampal neurogenesis^17^. Song et al. found GABA could directly affect NSCs in both local and distance regions^12,19^. The previous study has demonstrated that GABA inhibits proliferation of stem cells by means of GABAaR, the phosphatidylinositol-3-OH kinase (PI_3_K)-related kinase family and the histone variant H2AX^20,31^. In our work, we observed the inward current of RPCs induced by GABA treatment, the current caused the membrane depolarization (data not shown). In our previous work, the depolarization of NSCs led to the inhibition of proliferation^32^.

Therefore, our data has demonstrated that GABA could affect RPCs proliferation and self-renewal. Further, to depict the mechanism underlying growth inhibition, we examined possible changes in the expression levels of cell cycle regulators and found that GABA could up-regulate the p21, p27 and down-regulate Sox2 expression, which leads to the proliferation and self-renewal inhibition. The pluripotency factor Sox2 is an established regulator of neural and retinal precursor proliferation, self-renewal, and differentiation during development and is also required for maintenance of adult stem cell populations in many different tissues^33–35^. One recent study has identified that p21 acts as a transcriptional repressor of Sox2 in NSCs, p21 binds a Sox2 enhancer region to regulate Sox2 expression and adult neurogenesis, linking cell-cycle regulation with Sox2-mediated control of NSC expansion^36^.

Taken together, illustrating how the microenvironment affect the fate of RPCs helps us to explore new signaling pathway involved in the self-renewal and differentiation and develop a new strategy to improve the repair result after cell transplant into the degeneration retina.

## Materials and methods

### Isolation of RPCs and culture

Adult C57BL/6 mice of 1.5-months old were obtained from Beijing Vital River Laboratory Animal Technology Co., Ltd. Experimental procedures were approved by the Animal Care Committee of Huazhong University of Science and Technology and all experiments were performed in accordance with the relevant guidelines and regulations of Huazhong University of Science and Technology. Eyes were collected from as previously described with modification^37^. The retina was cut into small pieces, then enzymatically digested with collagenase I (10 mg/ml) and collagenase II (25 mg/ml) (Sigma, USA) for 10 min, centrifuged and discarded the supernatant, then re-suspended in culture medium for RPCs, which consisted DMEM/F12 medium (Lonza, USA) supplemented with murine basic fibroblast growth factor (bFGF, 20 ng/ml, PeproTech, USA), murine epidermal growth factor (EGF, 20 ng/ml, PeproTech, USA), B27 (1:50, Gibco, USA), N2 (1:100, Gibco, USA), insulin/transferrin/sodium selenite (1:500, Lonza, USA). Every 2 days, 2/3 medium was replaced by fresh medium. After 2 weeks of primary culture, very few spindle-shaped cells were observed, these cells expanded and formed colonies in the following 1–2 weeks. After the formation of this type of colony, Accutase (Sigma, USA) was used to digest these cells and they were further passaged, identified, and expanded. Cell differentiation assays were performed as previously described^37^.

### Immunostaining of cultured RPCs

Immunostaining of cultured cell was performed as described previously^32^. In general, RPCs were fixed with 4% formaldehyde (Sigma, USA) in PBS for 10 min at room temperature. After blocking with 10% goat serum, the cells were stained for 1 h at room temperature with one of the following primary antibodies: mouse anti-Nestin (1:400, Cat. Number: ab6142), mouse anti-Pax6 (1:500, Cat. Number: ab78545), mouse anti-Sox2 (1:300, Cat. Number: ab79351), rabbit anti-Chx10 (1:400, Cat. Number: ab133636), rabbit anti-Rax (1:300, Cat. Number: ab23340), mouse anti-Ki67 (1:400, Cat. Number: ab8191) (all primary anti-bodies purchased from Abcam, USA). The cells were then rinsed three times with PBS and incubated for 1 h at room temperature with the corresponding goat secondary antibody. Negative control for each fluorophore-conjugated secondary antibody, carried out without the addition of primary antibody, were included in order to evaluate nonspecific binding of secondary antibodies. After immunostaining, cells were counterstained with DAPI nuclear stain. All images were collected by a laser scanning spectral confocal microscope system (Leica, Germany).

### Real-time PCR

mRNA was extracted from RPCs and fetal mice retina with the Tryzol on the ice (Invitrogen, USA). First-strand cDNA synthesis was performed using a kit of cDNA synthesis (Invitrogen, USA). The transcript expression of each gene in RPCs was determined by normalizing to the (glyceraldehyde-3-phosphate dehydrogenase) GAPDH mRNA level. Real-time-PCR was performed using SYBR Green Master Mix (Bio-Rad, USA) with a Bio-Rad system (Bio-Rad, USA). The primers corresponding to the examined genes are as follows: Nestin: F 5′-CCTCAACCCTCACCACT CTATTTT-3′; R 5′-GCTTTTTACTGTCCCCGAGTT CTC-3′; Sox2: F 5′-TAGAG CTAGACTCCGGGCGAT GA-3′; R 5′-TTGCCTTAAACAAGACCACGAAA-3′; Pax6: F 5′-CACCACACCTGTCTCCTCCT-3′; R 5′-ATAACTCCGCCCATTCACT G-3′; Chx10: F 5′-GCCCACCTTCTTGGAAGTGCT-3′; R 5′-TGTGTCGCCGC TTCTTACGC-3′; Rax: F 5′-CCCTGAGGCTAAACTTGCAG-3′; R 5′-GTTCCCT TCTCCTCCTCCAC-3′; GAPDH: F 5′-ACGGCCGCATCTTCTTGTGCA-3′; R 5′-CAAGTG GGCCCCGGCCTTCTC-3′.

### Cell proliferation assay

To assess the cell growth curve, 50,000 cells were seeded in 35 mm dish coated with 0.1% gelatin (Sigma, USA). The cell number in each dish was counted with Coulter Counter (Beckman Coulter, USA) on days 3, 6, and 9. Cells treated without GABA were used as controls. The cell growth curve was calculated. Quantities of viable and nonviable cells were identified using the trypan blue exclusion assay.

### Flow cytometry for cell cycle analysis and apoptosis assay

RPCs were trypsinized and fixed by 70% ice-cold ethanol. RNase A (25 mg/ml) was used to treat cells for 30 min at 37 °C to eliminate RNA. Cells were stained with 50 mg/ml propidium iodide (PI) for 10 min at room temperature load to FACS Calibur flow cytometer (BD Biosciences, USA) to analysis of cell cycle. Data acquisition was performed using CellQuest software (BD Biosciences, USA), and the percentage of cells in the G_0_/G_1_, S, and G_2_/M phases was calculated using the Modfit. For apoptosis assay, cells were stained and analyzed with PI- and APC-conjugated Annexin V using an APC Annexin V Apoptosis kit (BD Biosciences, USA) according to the manufacturer’s protocol.

### Sphere formation

Neurosphere self-renewal assays were performed. Briefly, dissociated cells were plated at 1 viable cell per μl (1000 cells per well) in culture medium onto Ultra-Low attachment 6-well plate. Fresh medium was added to the culture dishes every other day. The total number of spheres that formed in each well was counted after a 7-day in vitro culture. Only phase bright live spheres containing at least 10 cells were counted. GABA was added to the medium, while the normal culture medium was set as control.

### Whole-cell patch-clamp recording

Glass coverslips plated with RPCs were transferred to a chamber containing the external solution (in mM: 125.0 NaCl, 2.5 KCl, 1.3 KH_2_PO_4_, 1.3 MgSO_4_, 25.0 NaHCO_3_, 2 CaCl_2_, 1.3 sodium L-ascorbate, 0.6 sodium pyruvate, 10 dextrose, pH 7.4, 320 mOsm), bubbled with 95% O_2_/5% CO_2_. Electrophysiological recordings were obtained at room temperature. RPCs were visualized by DIC microscopy. Microelectrodes (4–6 MΩ) were pulled from borosilicate glass capillaries and filled with the internal solution containing (in mM): 135 CsCl gluconate, 15 KCl, 4 MgCl_2_, 0.1 EGTA, 10.0 HEPES, 4 ATP magnesium salt, 0.3 GTP sodium salt, 7 phosphocreatine, pH 7.4, 300 mOsm. Data was collected using an Axon 200B amplifier and acquired with a DigiData 1322A (Axon Instruments) at 10 kHz. For measuring GABA-induced responses from RPCs, focal pressure ejection of 200 mM GABA or muscimol through a puffer pipette controlled by a Picospritze (2 s puff at 3–5 psi) was used to activate GABA_a_Rs under the whole-cell voltage-clamp. All drugs were purchased from Sigma except bicuculline (100 μM; Tocris, UK).

### Western-blot

For western blot analysis, proteins were separated in a 12% SDS-polyacrylamide gel. After electrophoresis, the gel was transferred onto PVDF membranes (Millipore, USA) and blocked for 3 h at 37 °C with 5% non-fat milk in Tris-buffered saline containing 0.1% Tween-20 (TBST). Primary antibodies were incubated at 4 °C overnight. Primary antibodies were used at the following dilutions: anti-p27 (Abcam, USA) at 1:2000, anti-p21 (Abcam, USA) at 1:2000, anti-p53 (Abcam, USA) and anti-phosphorylation of p53 at 1:2000, and anti-GAPDH (Abcam, USA) at 1:10,000. After primary antibody probing, membranes were washed in TBST, and incubated with HRP-conjugated secondary antibody (Dako, Denmark) at 1:5000 for 60 min at room temperature. After further washing, protein expression was detected by enhanced chemiluminescent (ECL) substrate (Pierce, Thermo Fisher Scientific, USA) and protein bands were visualized by film exposure. GAPDH was used as an internal control. The immunoreactive density was analyzed by Quantity One (Bio-Rad, USA).

### Statistical analyses

All quantitative values are presented as the mean ± SEM. Statistical comparisons were made using the unpaired Student’s *t* test with SPSS (version 15) software when appropriate. *p* < 0.05 was considered to be statistically significant.

## References

[1] Hartong DT, Berson EL, Dryja TP (2006). Retinitis pigmentosa. Lancet.

[2] Duong TT, Vasireddy V, Mills JA, Bennett J (2016). Retinas in a dish peek into inherited retinal degeneration. Cell Stem Cell.

[3] Sunness JS (2015). Stem cells in age-related macular degeneration and Stargardt’s macular dystrophy. Lancet.

[4] Garcia JM (2015). Stem cell therapy for retinal diseases. World J. Stem Cells.

[5] Cameron H, Tanapat P, Gould E (1997). Adrenal steroids and N-methyl-D-aspartate receptor activation regulate neurogenesis in the dentate gyrus of adult rats through a common pathway. Neuroscience.

[6] Seigel GM, Takahashi M, Adamus G, McDaniel T (1998). Intraocular transplantation of E1A-immortalized retinal precursor cells. Cell Transplant..

[7] Wan PX, Wang BW, Wang ZC (2015). Importance of the stem cell microenvironment for ophthalmological cell-based therapy. World J. Stem Cells.

[8] Alonso-Alonso ML, Srivastava GK (2015). Current focus of stem cell application in retinal repair. World J. Stem Cells.

[9] Marc RE, Liu W (2000). Fundamental GABAergic amacrine cell circuitries in the retina: nested feedback, concatenated inhibition, and axosomatic synapses. J. Comp. Neurol..

[10] Brandstätter JH, Hack I (2001). Localization of glutamate receptors at a complex synapse. Cell Tissue Res..

[11] Catavero C, Bao H, Song J (2018). Neural mechanisms underlying GABAergic regulation of adult hippocampal neurogenesis. Cell Tissue Res..

[12] Bao H (2017). Long-range GABAergic inputs regulate neural stem cell quiescence and control adult hippocampal neurogenesis. Cell Stem Cell.

[13] LoTurco JJ, Owens DF, Heath MJ, Davis MB, Kriegstein AR (1995). GABA and glutamate depolarize cortical progenitor cells and inhibit DNA synthesis. Neuron.

[14] Ben-Ari Y (2002). Excitatory actions of gaba during development: the nature of the nurture. Nat. Rev. Neurosci..

[15] Kemp PJ (2016). Improving and accelerating the differentiation and functional maturation of human stem cell-derived neurons: role of extracellular calcium and GABA. J. Physiol..

[16] Hsieh YC, Puche AC (2015). GABA modulation of SVZ-derived progenitor ventral cell migration. Dev. Neurobiol..

[17] Ming Gl, Song H (2011). Adult neurogenesis in the mammalian brain: significant answers and significant questions. Neuron.

[18] Young SZ (2012). NKCC1 knockdown decreases neuron production through GABAA-regulated neural progenitor proliferation and delays dendrite development. J. Neurosci..

[19] Song J (2012). Neuronal circuitry mechanism regulating adult quiescent neural stem-cell fate decision. Nature.

[20] Andang M (2008). Histone H2AX-dependent GABAA receptor regulation of stem cell proliferation. Nature.

[21] Yates D (2012). Neurogenesis: determining fate is a local activity. Nat. Rev. Neurosci..

[22] Chell JM, Frisen J (2012). Noisy neurons keep neural stem cells quiet. Cell Stem Cell.

[23] Du Y (2013). GABA exists as a negative regulator of cell proliferation in spermatogonial stem cells. Cell. Mol. Biol. Lett..

[24] Wagers AJ (2012). The stem cell niche in regenerative medicine. Cell Stem Cell.

[25] Sun W, Seigel GM, Salvi RJ (2002). Retinal precursor cells express functional ionotropic glutamate and GABA receptors. Neuroreport.

[26] Xie T, Li L (2007). Stem cells and their niche: an inseparable relationship. Development.

[27] Ben-Othman N (2017). Long-term GABA administration induces alpha cell-mediated beta-like cell neogenesis. Cell.

[28] Henschel O, Gipson KE, Bordey A (2008). GABAA receptors, anesthetics and anticonvulsants in brain development. CNS Neurol. Disord. Drug Targets.

[29] Young SZ, Bordey A (2009). GABA’s control of stem and cancer cell proliferation in adult neural and peripheral niches. Physiology.

[30] Giachino C (2014). GABA suppresses neurogenesis in the adult hippocampus through GABAB receptors. Development.

[31] Fernando RN (2011). Cell cycle restriction by histone H2AX limits proliferation of adult neural stem cells. Proc. Natl. Acad. Sci. U.S.A..

[32] Wang SJ, Weng CH, Xu HW, Zhao CJ, Yin ZQ (2014). Effect of optogenetic stimulus on the proliferation and cell cycle progression of neural stem cells. J. Membr. Biol..

[33] Sarkar A, Hochedlinger K (2013). The sox family of transcription factors: versatile regulators of stem and progenitor cell fate. Cell Stem Cell.

[34] Julian LM (2013). Opposing regulation of Sox2 by cell-cycle effectors E2f3a and E2f3b in neural stem cells. Cell Stem Cell.

[35] Surzenko N, Crowl T, Bachleda A, Langer L, Pevny L (2013). SOX2 maintains the quiescent progenitor cell state of postnatal retinal Muller glia. Development.

[36] Marques-Torrejon, M. A. et al. Cyclin-dependent kinase inhibitor p21 controls adult neural stem cell expansion by regulating Sox2 gene expression. *Cell Stem Cell*. 10.1016/j.stem.2012.12.001 (2012). 10.1016/j.stem.2012.12.001 PMC3714747 23260487

[37] Li, T., Lewallen, M., Chen, S., Yu, W., Zhang, N., Xie, T. Multipotent stem cells isolated from the adult mouse retina are capable of producing functional photoreceptor cells. Cell Res **23**, 788–802 (2013). 10.1038/cr.2013.48 PMC3674387 23567557

